# 40S ribosomal subunits scan mRNA for the start codon by one-dimensional diffusion

**DOI:** 10.1261/rna.080493.125

**Published:** 2025-10

**Authors:** Hironao Wakabayashi, Mingyi Zhu, Elizabeth J. Grayhack, David H. Mathews, Dmitri N. Ermolenko

**Affiliations:** Department of Biochemistry & Biophysics at the School of Medicine and Dentistry and Center for RNA Biology, University of Rochester, Rochester, New York 14642, USA

**Keywords:** mRNA scanning, one-dimensional diffusion, ribosome, translation initiation

## Abstract

During eukaryotic translation initiation, the small (40S) ribosomal subunit is recruited to the 5′ cap and subsequently scans the 5′ untranslated region (5′ UTR) of mRNA in search of the start codon. The molecular mechanism of mRNA scanning remains unclear, particularly the requirement for and identity of a translocase. Here, using GFP reporters in *Saccharomyces cerevisiae*, we show that order-of-magnitude variations in the length of unstructured 5′ UTRs have only modest effects on protein synthesis, whereas structured 5′ UTRs strongly inhibit translation. Thus, when not hindered by secondary structure, mRNA scanning is not rate limiting. Loss-of-function mutations in eIF4A, Ded1, and Slh1 reveal that these translational helicases are dispensable for mRNA scanning. Our data suggest that one-dimensional diffusion predominately enables 40S movement along the 5′ UTR during mRNA scanning.

## INTRODUCTION

In eukaryotes, translation typically begins at the AUG codon that is closest to the 5′ end of an mRNA (Kozak 1989b). During eukaryotic translation initiation, initiation factor eIF4E (eukaryotic Initiation Factor 4E) in complex with two other initiation factors, eIF4G and eIF4A, recognizes the m^7^Gppp cap structure at the 5′ end of the mRNA. The eIF4E•eIF4G•eIF4A complex, which is called eIF4F, recruits the small (40S) ribosomal subunit preassembled with initiator tRNA and initiation factors 1, 1A, 2, 3, and 5 (Aitken and Lorsch 2012). After recruitment to the 5′ end of mRNA, the small ribosomal subunit is believed to move along the 5′ UTR (Kozak and Shatkin 1978a,b; Kozak 1979; Archer et al. 2016) in search of the start codon.

mRNAs vary in their translational efficiencies by at least two orders of magnitude (Arava et al. 2003; Ingolia et al. 2009; Schwanhausser et al. 2011; Weinberg et al. 2016). The 5′ UTR is thought to play a critical role in regulating translation initiation through a variety of mechanisms, many of which are not well understood. Although 5′ UTR sequence and secondary structure are involved in translational control, the mechanism by which the 40S subunit moves along the 5′ UTR and the regulation of 40S scanning by the 5′ UTR remain obscure. It is commonly assumed that scanning is driven by one of the core initiation factors, DEAD-box RNA helicases eIF4A (Shirokikh and Preiss 2018). eIF4A is thought to either translocate along mRNA (Garcia-Garcia et al. 2015) in a processive manner or bias diffusion of the 40S toward the start codon in an ATPase-dependent manner (Spirin 2009). The latter idea is often referred to as the Brownian ratchet model of scanning (Spirin 2009; Gu et al. 2021). Other translational helicases, such as Ded1 (DDX3 in mammals), are thought to contribute to 40S scanning through structured 5′ UTRs (Gao et al. 2016; Gupta et al. 2018). Helicase-driven models of 40S scanning tend to equate secondary structure unwinding and 40S movement along the 5′ UTR although these may be mechanistically distinct processes. 40S migration along the 5′ UTR could also occur by factor-independent diffusion (Pestova and Kolupaeva 2002; Chappell et al. 2006; Paek et al. 2015; Li et al. 2022).

The relationship between 5′ UTR length and translation should provide critical insights into the mechanism of scanning. However, entanglement of secondary structure unwinding and 40S movement along the 5′ UTR also hampers understanding regulation of 40S scanning by 5′ UTR length. When compared to ORFs and 3′ UTRs, the length of 5′ UTRs is relatively short in diverse taxonomic classes of eukaryotes, providing indirect evidence that 5′ UTR scanning by the 40S may be rate-limiting in translation (Lin and Li 2012; Leppek et al. 2018). For example, median 5′ UTR lengths in yeast and human cells are ∼50 and 200 nt, respectively (Leppek et al. 2018). Consistent with mRNA scanning being rate-limiting in translation, computational analysis revealed negative correlation between protein expression and 5′ UTR length (Lin and Li 2012). Translation of reporter mRNAs performed in cell extracts also showed that lengthening of the 5′ UTR progressively reduced translation (Berthelot et al. 2004; Vassilenko et al. 2011). In contrast, experiments in yeast cells (Berthelot et al. 2004; Sen et al. 2015), *Xenopus* oocytes (Gallie et al. 2000), and mammalian cell extracts (Kozak 1991) showed that translation does not depend on the length of the 5′ UTR (Berthelot et al. 2004; Sen et al. 2015) or is enhanced by lengthening of the 5′ UTR (Kozak 1991; Gallie et al. 2000). The discrepancy between different studies might stem from the use of different translation systems as well as superimposition of the effects of 5′ UTR length and secondary structure.

To reexamine how the 5′ UTR length affects translation, we use intrinsically unstructured, nonrepetitive sequences that are significantly longer than repetitive unstructured 5′ UTRs used in previous studies (Pestova and Kolupaeva 2002; Sen et al. 2015). We find that extending the 5′ UTR of the GFP reporter 10-fold over the median yeast 5′ UTR length moderately reduces translational efficiency in yeast cells. We also find that loss-of-function mutations or deletions of translational helicases eIF4A, Ded1, and Slh1 similarly affect reporter mRNAs with short and long 5′ UTRs, indicating that these helicases are not rate-limiting for 40S movement along unstructured 5′ UTRs. Based on these observations, we hypothesize that, at least in yeast, 40S movement along the 5′ UTR might be predominantly driven by one-dimensional diffusion, rather than by a translocase.

## RESULTS

### When 40S scanning is rate-limiting, mRNAs with longer 5′ UTRs are expected to be translated less efficiently than mRNAs with shorter 5′ UTRs

To analyze putative experimental outcomes of examining dependence of protein synthesis on 5′ UTR length using GFP reporters, we considered a simple mathematical approximation: The amount of translated protein is inversely proportional to the time needed to synthesize one GFP molecule. The time needed to synthesize one GFP molecule accounts for the duration of 40S scanning, which depends on the length of the 5′ UTR (*L*), and sum of all other steps of initiation, elongation, and termination, which do not depend on the 5′ UTR length ($\sum_{\;j=1}^{N}\tau_{j}$ in Eqs. 1 and 2). In this simplified approximation, mRNA half-lives and GFP protein degradation rates are assumed to be constant, independent of 5′ UTR length and unaffected by the overall rate of GFP synthesis. The duration of 40S scanning should scale linearly with 5′ UTR length if scanning is driven by a translocase/helicase (Vassilenko et al. 2011):

(1) $$[\mathrm{GFP}]\propto \frac{T}{\left(L÷k_{\mathrm{tr}}+\sum_{\;j=1}^{N}\tau_{j}\right)},$$

where *T* = total time, *L* = length of the 5′ UTR, *k*_tr_ = rate of 40S translation along the 5′ UTR, and τ_j_ = duration of *N* translation steps other than 40S scanning, including other steps of initiation such as 40S recruitment that are independent of 5′ UTR length.

Alternatively, scanning time should scale as the square of 5′ UTR length if scanning occurs by diffusion (Vassilenko et al. 2011):

(2) $$[\mathrm{GFP}]\propto \frac{T}{\left(L^{2}÷D+\sum_{\;j=1}^{N}\tau_{j}\right)},$$

where *D* = 40S diffusion coefficient.

**FIGURE 1.**
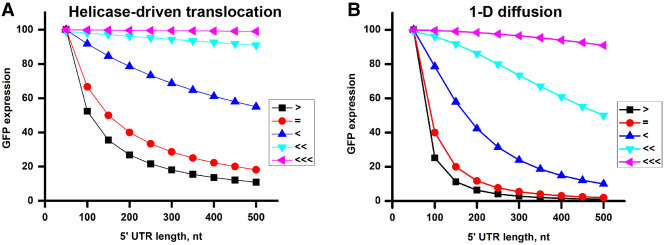
Modeled dependencies of GFP synthesis on the length of the 5′ UTR for the helicase-driven (*A*, Eq. 1) and 1D diffusion (*B*, Eq. 2) mechanisms of 40S scanning. Amounts of synthesized GFP are inversely proportional to 5′ UTR scanning time and sum of all other steps of initiation, elongation, and termination, which do not depend on the 5′ UTR length. GFP synthesis is modeled for scenarios when the sum of all other steps of translation, which are independent of 5′ UTR length, are 10-fold faster (> black), equal to (= red), 10-fold slower (< blue), 100-fold slower (<< cyan), or 1000-fold slower (<<< magenta) than the scanning of the 50 nt-long 5′ UTR.

The reciprocal relationship between the amount of synthesized protein and 5′ UTR length is expected for both translocation and diffusional mechanism of scanning (Fig. 1). In the experiments reported in this manuscript involving variation in 5′ UTR length, the term $\sum_{\;j=1}^{N}\tau_{j}$ in Equations 1 and 2 is expected to be a constant.

Both translocation and 1D diffusion models predict that if scanning is much faster than the sum of all other steps of protein synthesis, including other steps of initiation, protein synthesis is expected to be nearly independent of 5′ UTR length (Fig. 1). Conversely, in the case of 40S scanning being comparable to or even slower than the sum of all other steps of translation, protein synthesis is expected to show strong dependence on the 5′ UTR length (Fig. 1). Under these conditions, it might be possible to distinguish between translocation and diffusion mechanisms of scanning by fitting the dependence of GFP synthesis on the 5′ UTR length to Equations 1 and 2. However, this may be challenging given similarities in shapes of translocation and 1D curves (Fig. 1).

Another prediction that can be inferred from these simplified models is that a reduction of scanning rate (e.g., via mutation) is expected to more significantly affect translation of mRNAs with longer 5′ UTRs. On the contrary, slowing down any other step of translation is expected to have a stronger relative effect on translation of mRNAs with shorter 5′ UTRs because the scanning-independent steps of translation make larger contributions to the time needed to synthesize one GFP molecule on mRNAs with shorter 5′ UTRs than on mRNAs with longer 5′ UTRs.

### Lengthening of the 5′ UTR through addition of UCC/ACCAC inhibitory sequences substantially reduces translation

**FIGURE 2.**
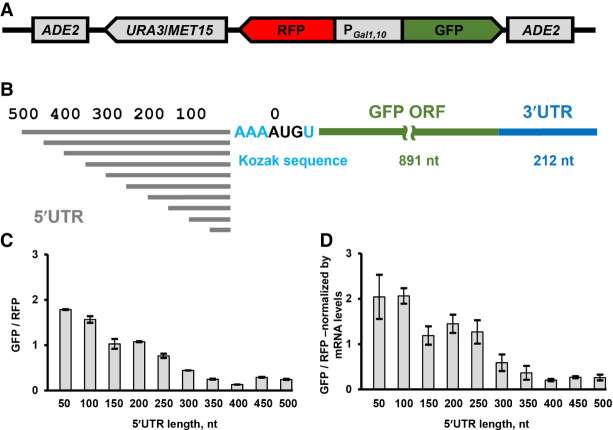
Lengthening unstructured 5′ UTR enriched with UCC and ACCAC sequences progressively reduces translation of GFP reporter in yeast cells. (*A*) Schematic depiction of RNA-ID reporter that includes *ADE2* loci for integration into the yeast genome, *URA3* or *MET15* selection marker gene (details in Materials and Methods), *Gal 1,10* bidirectional promoter, RFP, and sfGFP ORFs. (*B*) Design of GFP mRNAs containing unstructured 5′ UTRs (NUS′) of different lengths. The constructs share the same NUS sequence but differ in how much of the NUS sequence is included from the 5′ end. (*C*) Mean GFP/RFP fluorescence ratios measured in yeast cells, which were transformed with different RNA-ID 5′ UTR NUS′ constructs as indicated. GFP/RFP ratio in GO RNA-ID with 6 nt-long 5′ UTR was set to 1. Spearman rank correlation coefficient *R* (Virtanen et al. 2020) between length and GFP/RFP ratio was determined to be −0.915 (*P* < 0.0001). (*D*) Mean GFP/RFP fluorescence ratios normalized to the ratio of GFP and RFP mRNA levels, which were determined by RT-qPCR. Error bars show standard deviations determined from three biological replicates.

To experimentally test how the length and secondary structure of the 5′ UTR affect translation and which protein factors are critical for scanning in live cells, we used the RNA-ID reporter system (Fig. 2A; Dean and Grayhack 2012). The RNA-ID reporter provides a robust and sensitive way to examine the effects of *cis*-regularity mRNA sequences on translation in yeast cells. The RNA-ID DNA construct contains the ORFs of superfolder GFP and the yeast codon-optimized red fluorescent protein variant of mCherry under control of the bidirectional *GAL1,10* promoter, with the *MET15* or *URA3* gene used for selection in yeast, and flanking sequences allowing for the integration into the *ADE2* locus of the yeast genome via homologous recombination. GFP levels are measured by GFP fluorescence normalized by the fluorescence of RFP to account for differential activation of the promoter in different cells.

In all RNA-ID constructs, the ORF of superfolder GFP was flanked by the 3′ UTR from yeast *RPL41B* mRNA (Fig. 2A). *RPL41B* mRNA is one of the most well-translated mRNAs in yeast cells; the 3′ UTRs of this mRNA were previously precisely identified using rapid amplification of cDNA ends (RACE) (Yu and Warner 2001). To examine the effect of the 5′ UTR length on translation, upstream of the GFP ORF, we introduced intrinsically unstructured sequences ranging from 50 to 500 nt in length in 50 nt increments at the 5′ end of the 5′ UTR while preserving the original Kozak context of the start codon (Fig. 2B; Supplemental Table S1). These nonrepetitive unstructured sequences (NUS) were designed with the previously developed genetic algorithm (Lai et al. 2018). The genetic algorithm evolves a sequence through an iterative sequence alteration/selection process that eliminates all alternative base-pairing interactions, including those pairs that the 5′ UTR nucleotides can form with the entire mRNA sequence including the ORF and 3′ UTR. A single 500 nt NUS was designed, and individual constructs using differing lengths were created by truncation of the 5′ end. Therefore, these constructs share the sequence upstream of the Kozak sequence and the start codon. Importantly, in contrast to CA and CAA repeats commonly used to eliminate RNA secondary structure, NUSs are more complex and more easily propagated in *Escherichia coli* and yeast cells. Base-pairing probabilities estimated by the RNAstructure software package predict that NUS nucleotides are nearly completely unpaired while most nucleotides in the ORF and 3′ UTR have high (>75%) base-pairing probability (Supplemental Fig. S1).

Resulting RNA-ID constructs (named ^50^NUS′-^500^NUS′ where superscript numbers indicate 5′ UTR length) were transformed into BY4741 haploid strain of *Saccharomyces cerevisiae*. GFP/RFP ratio for each construct was measured by flow cytometry (Supplemental Fig. S2) and normalized to GFP/RFP levels observed in the RNA-ID GO control strain, in which the GFP mRNA contains a 6 nt-long 5′ UTR (Supplemental Table S1). Extending the 5′ UTR progressively reduced GFP synthesis until it leveled off at the length of 350–400 nt (Fig. 2C). Normalizing GFP/RFP fluorescence by the GFP/RFP ratio of mRNA levels, which were determined by RT-qPCR, indicated that the observed reduction in GFP synthesis was due to a decrease in translational efficiency rather than changes in mRNA levels (Fig. 2D). Translation of GFP mRNAs with 350–500 nt-long unstructured 5′ UTRs was approximately eight times lower on average than the translation of mRNA with a 50 nt-long 5′ UTR. These data showed considerable dependence of translation on the 5′ UTR length, which could be due to either length-dependent or sequence-specific effects. Indeed, further analysis of ^50^NUS′-^500^NUS′ sequences revealed that extending the 5′ UTR from 50 to 500 nt increasingly enriched NUS sequences with C-rich motifs UCC and ACCAC (Supplemental Fig. S3). These C-rich sequences were recently reported to be inhibitory for translation initiation (Niederer et al. 2022).

### Order-of-magnitude variations in the length of unstructured 5′ UTR depleted of UCC/ACCAC inhibitory sequences have only modest effects on translation in yeast cells

**FIGURE 3.**
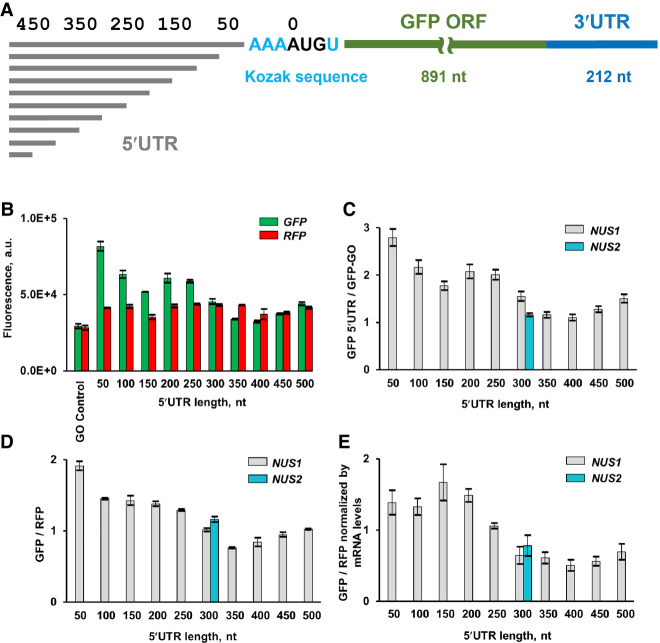
Tenfold lengthening of unstructured 5′ UTR moderately reduces translation of GFP reporter in yeast cells. (*A*) Design of GFP mRNAs containing unstructured 5′ UTRs (NUS1 series) of different lengths. (*B*) Mean GFP and RFP fluorescence measured in yeast cells, which were transformed with different RNA-ID constructs as indicated. (*C*) Mean GFP fluorescence in strains expressing RNA-ID mRNA with unstructured 5′ UTRs of different lengths normalized by GFP fluorescence produced in the GO RNA-ID control reporter with a 6 nt-long 5′ UTR. (*D*) Mean GFP/RFP fluorescence ratios. GFP/RFP ratio in the GO RNA-ID control was set to 1. Spearman rank correlation coefficient *R* (Virtanen et al. 2020) between length and GFP/RFP ratio was determined to be −0.841 (*P* < 0.0001). (*E*) Mean GFP/RFP fluorescence ratios normalized to the ratio of GFP and RFP mRNA levels, which were determined by RT-qPCR. NUS1 and NUS2 are independently designed, divergent, nonrepetitive, unstructured sequences. Error bars show standard deviations determined from three biological replicates.

To test whether observed reduction of protein synthesis in ^50^NUS′-^500^NUS′ constructs was due to the presence of inhibitory C-rich sequences or increased 5′ UTR length, we computationally designed a new set of unstructured 5′ UTRs (^50^NUS1-^500^NUS1) ranging in length from 50 to 500 nt, in which UCC and ACCAC sequence motifs were avoided (Supplemental Table S1). Because sequences adjacent to the 5′ cap may influence 40S recruitment, we extended NUS1 UTRs with 50 nt increments at the 3′ end while keeping the 5′ end constant (Fig. 3A). As expected, RFP fluorescence remained nearly constant between strains transformed with different RNA-ID NUS1 constructs while extending the 5′ UTR in GFP mRNA moderately reduced GFP synthesis (Fig. 3B–D). Transforming ^50^NUS1-^500^NUS1 constructs into the BY4741 and BY4742 strains produced similar results (Supplemental Fig. S4). Translation of ^50^NUS1 was only twofold more efficient than translation of ^500^NUS1 (Fig. 3C–E). Hence, the larger length-dependent reduction of translational efficiency observed in ^50^NUS′-^500^NUS′ constructs seems likely to be due to the presence of UCC/ACCAC inhibitory sequences or, possibly, to effects of 40S recruitment caused by varying the sequence adjacent to the 5′ cap. In any case, with the NUS1 constructs, translation efficiency is relatively independent of the 5′ UTR length.

To further explore how sequence variations in the unstructured 5′ UTR affect translation, we independently designed another 300 nt-long 5′ UTR (^300^NUS2), in which UCC and ACCAC sequences were also avoided. ^300^NUS1 and ^300^NUS2 shared only 57.3% identity in a global pairwise alignment calculated by the EMBOSS Stretcher alignment tool and 58.8% identity in a local alignment calculated by the EMBOSS Water alignment tool (Madeira et al. 2019). Similar sequence identity (mean of 56.9% and standard deviation 2.1%) was found in the local alignment of 100 computationally shuffled (i.e., randomized) ^300^NUS1 and ^300^NUS2 sequences. GFP/RFP ratio in ^300^NUS1 and ^300^NUS2 were similar (Fig. 3C–E), indicating that as long as inhibitory motifs were eliminated from the design, other sequence variations had minimal effect on translation. We thus focused the rest of our experiments on the ^50^NUS1-^500^NUS1 constructs.

Normalizing GFP/RFP ratio by mRNA levels, which were determined by RT-qPCR, showed that observed changes in GFP synthesis in ^50^NUS1-^500^NUS1 strains were due to modest reductions of translational efficiency rather than changes in mRNA levels (Fig. 3E). It is noteworthy that GFP/RFP ratios (Fig. 3D,E) and GFP levels alone (Fig. 3C) showed similar dependencies on 5′ UTR length. Hence, the transcriptional activity of the *Gal1,10* promoter is effectively constant in different cells, and GFP fluorescence alone (without normalizing to RFP levels) can be reliably used to analyze changes in translational efficiency of GFP reporter constructs.

To test whether in yeast cells, ^50^NUS1-^500^NUS1 mRNAs are transcribed from the previously identified transcription start site of the *GAL1,10* promoter, we performed 5′ RACE analysis for ^50^NUS1, ^300^NUS1, and ^500^NUS1 mRNAs. 5′ RACE PCR products were of expected size and sequence (Supplemental Fig. S5), consistent with the predicted transcription start. SDS-PAGE analysis combined with GFP fluorescence imaging revealed a single fluorescent GFP protein band in all ^50^NUS1-^500^NUS1 strains, indicating that only one ORF, which produced fluorescent GFP, was predominately translated (Supplemental Fig. S6). Hence, no alternative near-cognate, in-frame start sites were used for translation initiation in ^50^NUS1-^500^NUS1 GFP mRNAs.

### RNA secondary structures in the 5′ UTR strongly inhibit translation

**FIGURE 4.**
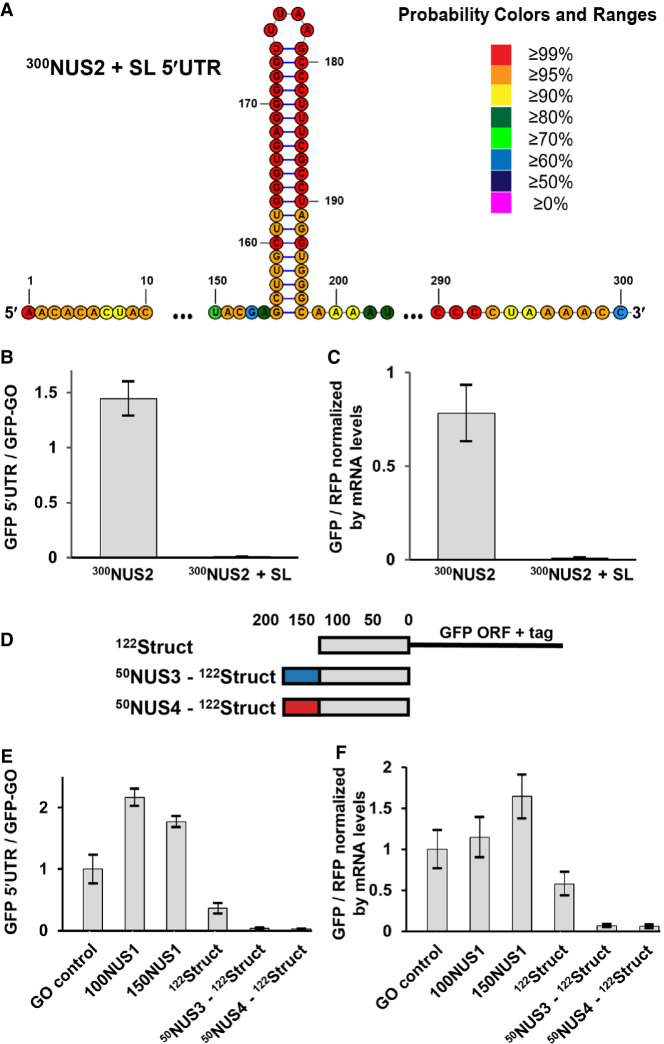
RNA secondary structure in the 5′ UTR strongly inhibits translation. (*A*) Fifty-four nucleotides in the middle of ^300^NUS2 5′UTR were replaced with a 20 bp-long RNA stem–loop to create ^300^NUS2 + SL mRNA. Base-pair probabilities determined by the RNAstructure software are shown by color code as indicated. (*B*,*C*) Introducing the stem–loop (SL) into the ^300^NUS2 5′ UTR reduced GFP synthesis. (*D*–*F*) A 122 nt-long sequence (^122^Struct) in the 5′ UTR (gray box in schematics *D*), which is predicted to fold into multiple stem–loops, inhibits GFP synthesis. (*B*,*E*) Mean GFP fluorescence was normalized to that in the GO RNA-ID reporter with a 6 nt-long 5′ UTR. (*C*,*F*) Mean GFP/RFP fluorescence ratios normalized to the ratio of GFP and RFP mRNA levels, which were determined by RT-qPCR. GFP/RFP ratio in the GO RNA-ID control was set to 1. Error bars (*B*,*C*,*E*,*F*) show standard deviations determined from three biological replicates.

Our experiments with ^50^NUS1-^500^NUS1 reporter constructs showed that extending the 5′ UTR 10-fold over the median length of yeast 5′ UTRs only modestly reduces protein synthesis. Therefore, 40S scanning along an unstructured 5′ UTR seems quite effective and does not hamper translation initiation. We next explored the effects of 5′ UTR secondary structure on translational efficiency and 40S scanning. To that end, we replaced nucleotides 156–199 of ^300^NUS2 5′ UTR with a GC-rich stem–loop containing 20 bp and a UUAA tetraloop (Fig. 4A). When compared to the ^300^NUS2 5′ UTR, the ^300^NUS2 5′ UTR with the cap distal stem–loop (^300^NUS2-SL) produced ∼150-fold less GFP (Fig. 4B). When adjusted by mRNA levels measured by RT-qPCR, the stem–loop reduced GFP translation by 85-fold (Fig. 4C). Likewise, we replaced 44 nt 150 nt downstream from the 5′ end of ^300^NUS′ and ^500^NUS′ 5′ UTRs with a cap-distal stem–loop containing 20 bp and a tetraloop. The insertion of this stem–loop into ^300^NUS′ and ^500^NUS′ 5′ UTRs inhibited GFP synthesis by 30- and 10-fold, respectively (Supplemental Fig. S7). Hence, consistent with many published reports (Pelletier and Sonenberg 1985; Kozak 1986, 1989a; Vega Laso et al. 1993; Babendure et al. 2006; Sen et al. 2015), stable RNA secondary structure strongly inhibits translation, presumably by hindering 40S scanning. These results also indicate that ^50^NUS-^500^NUS (as well as ^50^NUS′-^500^NUS′) mRNA constructs are translated in a cap- and scanning-dependent manner rather than through a noncanonical, cap-independent mechanism (e.g., IRES-dependent initiation).

Next, instead of introducing a very stable stem–loop into an unstructured 5′ UTR, we replaced the entire 5′ UTR with a 122 nt-long sequence that is derived from the 5′ UTR of human GAPDH mRNA to make the ^122^Struct construct. The RNAstructure software package predicts that most nucleotides of this sequence have between 50% and 75% probability of base-pairing (Supplemental Figs. S8, S9). Rather than forming one very stable secondary structure, the ^122^Struct sequence likely folds into a dynamic ensemble of alternative structures. When compared to the ^100^NUS1 and ^150^NUS1 strains, the ^122^Struct strain produced five- and sixfold less GFP, respectively (Fig. 4E). The GFP/RFP ratio normalized to mRNA levels in ^122^Struct strain was two- and threefold lower than that in the ^100^NUS1 and ^150^NUS1 strains, respectively (Fig. 4F), indicating that a structured 5′ UTR substantially reduces translation relative to an unstructured 5′ UTR of similar length.

Because the ^122^Struct 5′ UTR is predicted to contain base pairs near the 5′ end (Supplemental Figs. S8, S9), secondary structures of the ^122^Struct 5′ UTR may inhibit both initial 40S recruitment and following 40S scanning (Babendure et al. 2006). We next aimed to eliminate the effects of secondary structure on 40S recruitment. As the 43S initiation complex covers ∼45 nt of mRNA (Kozak and Shatkin 1977; Brito Querido et al. 2020), we added 50 nt-long unstructured sequences NUS3 or NUS4 upstream of the ^122^Struct sequence that would allow for unhindered 40S binding. Surprisingly, adding the NUS3 or NUS4 unstructured sequence reduced GFP levels 10- or 14-fold, respectively, relative to GFP levels in the ^122^Struct 5′ UTR strain (Fig. 4E). GFP/RFP ratios normalized to mRNA levels in NUS3-^122^Struct and NUS4-^122^Struct strains were ninefold lower than that in the ^122^Struct 5′ UTR strain (Fig. 4F). One possible explanation of these results is that 40S binding to the original ^122^Struct mRNA partially unwound secondary structures of the 5′ UTR and thus aided 40S scanning. 40S recruitment to the unstructured sequence (NUS3 or NUS4) upstream of the ^122^Struct preserved all secondary structure elements within the ^122^Struct sequence and thus reduced effectiveness of 40S scanning on NUS3–^122^Struct and NUS4–^122^Struct mRNAs. Taken together, experiments with the ^300^NUS2-SL and ^122^Struct mRNAs demonstrate that secondary structure elements within the 5′ UTR strongly inhibit protein synthesis by impeding 40S scanning.

### Translational helicase eIF4A is not involved in 40S movement along the 5′ UTR

**FIGURE 5.**
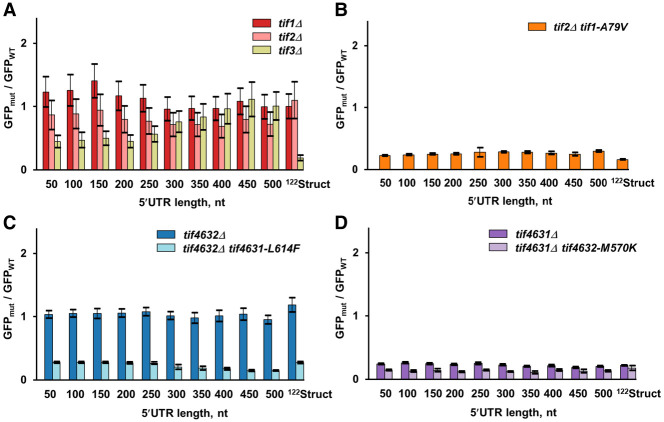
Mutational perturbations of eIF4F subunits similarly reduce translation of GFP NUS1 mRNAs with short and long unstructured 5′ UTRs. GFP fluorescence measured in mutant strains normalized to respective GFP fluorescence measured in the wild-type strain. Error bars indicate standard deviations determined from three biological replicates. (*A*) eIF4A-encoding *tif1*, *tif2* or eIF4B-encoding *tif3* were deleted in BY4741 background as indicated. (*B*) Either wild-type (*TIF1*) or temperature-sensitive (*tif1-A79V*) alleles of the eIF4A-encoding *tif1* were expressed in the strain lacking both chromosomal copies of *tif1* and *tif2*. (*C*) An amino acid substitution L614F, which reduces eIF4G binding to eIF4A, was introduced into *tif4631* in the background of *tif4632* deletion to create the temperature-sensitive *tif4632*Δ*tif4631-L614F* strain. (*D*) An amino acid substitution M570K, which reduces eIF4G binding to eIF4A, was introduced into *tif4632* in the background of a *tif4631* deletion to create temperature-sensitive *tif4631*Δ*tif4632-M570K* strain. BY4741 strain is considered as wild type in *A*, *C*, and *D*. In experiments with temperature-sensitive strains *tif2*Δ*tif1-A79V* and *tif4632*Δ*tif4631-L614F*, the *Gal 1,10* promoter was induced concurrently with raising the incubation temperature from 30°C to 37°C. Respective wild-type control strains received identical treatment.

Slowing 40S scanning is expected to more substantially affect mRNAs with longer 5′ UTRs (Fig. 1). We next used mutants to test whether mRNAs with long unstructured 5′ UTRs are particularly sensitive to genetic perturbations of translation factors, which were previously implicated in 40S scanning. DEAD-box helicase eIF4A is a subunit of the 5′ cap recognition complex eIF4F. The helicase activity of eIF4A is thought to be directly involved in ATP-dependent 40S movement along the 5′ UTR (Kozak 1980; Spirin 2009; Brito Querido et al. 2024). In yeast, eIF4A is encoded by two paralogs *TIF1* and *TIF2*. To test involvement of eIF4A in 40S scanning, RNA-ID constructs were transformed into either *tif1*Δ or *tif2*Δ deletion strains created in a BY4741 genetic background. RT-qPCR measurements showed an approximately twofold decrease in eIF4A mRNA levels in both *tif1*Δ and *tif2*Δ strains when compared to the parent (wild-type) BY4741 strain (Supplemental Fig. S10). Nevertheless, GFP synthesis on the ^50-500^NUS1 and ^122^Struct mRNAs was essentially unaffected by either *tif1* or *tif2* deletion (Fig. 5A). Likewise, GFP synthesis on the ^50-500^NUS′ mRNAs was unchanged in *tif1*Δ or *tif2*Δ strains (Supplemental Fig. S11A). This could be due to high expression levels of eIF4A, which is the most abundant factor of translation initiation in yeast cells (Kulak et al. 2014). Consistent with the observation that protein synthesis was unaffected by the deletion of either *tif1* or *tif2*, growth of *tif1*Δ or *tif2*Δ strains on YPD plates was similar to the parental, wild-type (BY4741) strain (Supplemental Fig. S12).

Because eIF4A is essential for yeast viability, we could not delete both *TIF1* and *TIF2* genes simultaneously. Instead, we integrated RNA-ID constructs into the strain lacking both *TIF1* and *TIF2* genes and expressing the temperature-sensitive allele *tif1-A79V* (Sen et al. 2015). A79V substitution is thought to weaken eIF4A binding to RNA (Schmid and Linder 1991; Linder and Jankowsky 2011; Sen et al. 2015) and severely hamper yeast growth at 37°C (Supplemental Fig. S13). To examine the effects of the temperature-sensitive A79V mutation in eIF4A, GFP expression was induced by the addition of galactose concurrently with switching from 30°C to 37°C. In the absence of galactose, negligible levels of GFP were accumulated, indicating tight transcriptional control of the *Gal 1,10* promoter (Supplemental Fig. S14). When compared at 37°C to the strain in which the wild-type *TIF1* allele was expressed, expressing *tif1-V79A* decreased GFP levels fourfold in all ^50-500^NUS1 constructs irrespective of 5′ UTR length (Fig. 5B). Similar uniform reduction of GFP synthesis was observed for all the ^50-500^NUS′ mRNAs (Supplemental Fig. S11B). Translation on the ^122^Struct mRNA containing a 122 nt-long structured 5′ UTR was reduced sixfold, that is, fairly similar to translation of mRNAs with unstructured 5′ UTRs (Fig. 5B). This result is consistent with published data indicating that eIF4A stimulates translation of mRNAs regardless of the structural complexity of their 5′ UTR (Yourik et al. 2017).

Helicase activity of eIF4A is stimulated by another initiation factor, eIF4B, which is encoded by a single-copy gene *TIF3*. We next deleted the nonessential gene *TIF3* in a BY4741 background. In the *tif3*Δ strain, translation of GFP mRNAs with 50–200 nt-long unstructured 5′ UTRs was reduced twofold, while translation of GFP mRNAs with 300–500 nt-long unstructured 5′ UTRs was unaffected (Fig. 5A). Similar results were obtained with the ^50-500^NUS′ reporters (Supplemental Fig. 11A). Higher sensitivity of mRNAs with shorter 5′ UTRs to *TIF3* deletion may indicate involvement of this protein in the 40S recruitment step of translation initiation (Eqs. 1 and 2). Remarkably, the ^122^Struct mRNA, containing a 122 nt-long structured 5′ UTR, was far more sensitive to eIF4B deletion than mRNAs with unstructured 5′ UTRs as GFP levels decreased sixfold in the *tif3*Δ strain. This observation agrees with ribosome profiling data showing that eIF4B stimulates translation of mRNAs with structured 5′ UTRs (Sen et al. 2016).

eIF4A helicase and ATPase activities are stimulated by interaction with another subunit of the eIF4F complex, eIF4G (Rogers et al. 2002; Schutz et al. 2008; Rajagopal et al. 2012). In yeast, eIF4G is encoded by two paralogs, *TIF4631* and *TIF4632.* When we deleted the *TIF4632* gene, translation of all RNA-ID constructs was unaffected (Fig. 5C). We next introduced an amino acid substitution L614F into *tif4631* in the presence of *tif4632*Δ. The L614F mutation weakens the interaction of eIF4G with eIF4A and produces a temperature-sensitive phenotype, which can be suppressed by overexpression of eIF4A or eIF4B (Supplemental Fig. S13; Park et al. 2013). When measured at 37°C, the L614F mutation in *tif4631* reduced GFP synthesis of the ^50^NUS1, ^500^NUS1, and ^122^Struct mRNAs by four-, seven- and fourfold, respectively (Fig. 5C). Relative to *tif4632*Δ strain, GFP levels in *tif4632*Δ *tif4631-L614F* strain transformed with either the ^50^NUS′ or ^500^NUS′ RNA-ID reporter were equally reduced by threefold (Supplemental Fig. 11C).

Because mRNAs with longer NUS1 5′ UTRs were slightly more sensitive to *tif4631-L614F* mutation than mRNAs with shorter 5′ UTRs, we also made reciprocal mutational perturbations in *tif4632* and *tif4631* to further examine involvement of eIF4A–eIF4G interaction in scanning. In that, we deleted *tif4631* and introduced an amino acid substitution M570K, which weakens the interaction of eIF4A with eIF4G (Park et al. 2013), into *tif4632.* Deletion of *tif4631* lowered GFP levels fivefold irrespective of 5′ UTR length in both the ^50-500^NUS1 and ^50-500^NUS′ reporters (Fig. 5D; Supplemental Fig. S11D). Mutation M570K in *tif4632* further reduced GFP levels 1.5- to twofold in all 5′ UTR NUS1 and NUS′ reporters (Fig. 5D; Supplemental Fig. S11D). Relative to the *tif4631*Δ strain, translation of the ^122^Struct mRNA decreased by 1.2-fold in the *tif4631*Δ *tif4632-M570K* strain (Fig. 5D).

Taken together, our results indicate that amino acid substitutions disrupting the eIF4A–eIF4G interaction similarly affected GFP reporters with short and long 5′ UTRs. Hence, the eIF4F complex as well as eIF4A alone are unlikely to be responsible for 40S movement along the 5′ UTR.

### Loss-of-function mutations in eIF3g and eIF3i moderately affect translation of mRNAs with short and long unstructured 5′ UTRs

We next probed whether initiation factors eIF3g and eIF3i, which are encoded by yeast genes *TIF35* and *TIF34*, respectively, affect 40S scanning. These two subunits of multimeric initiation factor 3 bind near the mRNA entry site of the 40S subunit, that is, the “front” side of the small subunit in respect to the direction of scanning. eIF3g and eIF3i were shown to stimulate ATPase activity of eIF4A (Yourik et al. 2017). In a recent cryo-EM reconstruction of the 48S initiation complex, two eIF4A molecules were visualized. One was bound at the mRNA entry site; the other was bound to the eIF4G•eIF4E•5′cap near the “rear” side of the small subunit (Brito Querido et al. 2024). eIF3i was seen making extensive interactions with eIF4A bound to the mRNA entry site, providing further evidence for possible eIF3i involvement in helicase and translocase activities of the 43S initiation complex.

**FIGURE 6.**
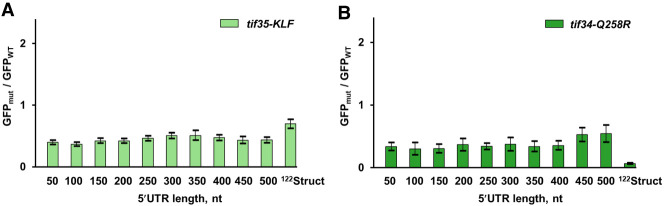
Mutations in eIF3g and eIF3i similarly reduce translation of GFP NUS1 mRNAs with short and long unstructured 5′ UTRs. Amino acid substitutions were introduced in eIF3g-encoding *tif35* (*A*) or eIF3i-encoding *tif34* (*B*) in the BY4741 genetic background. GFP fluorescence measured in mutant strains was normalized to the respective GFP fluorescence measured in the wild-type (BY4741) strain. Error bars indicate standard deviations determined from three biological replicates. In experiments with the temperature-sensitive *tif35-KLF* strain*,* the *Gal 1,10* promoter was induced concurrently with raising the incubation temperature from 30°C to 37°C for both *tif35-KLF* and wild-type cells.

In yeast, triple alanine substitution K194A/L235A/F237A of conserved residues in the RRM of eIF3g (*tif35-KLF*) or a single-point mutation Q258R in the WD40 repeat 6 of eIF3i (*tif34-Q258R*) was shown to produce growth defects (Cuchalova et al. 2010). These mutations also affected the efficiency of reinitiation on the *GCN4* mRNA, which contains four short uORFs upstream of the main ORF (Cuchalova et al. 2010). Based on these observations, it was hypothesized that eIF3g and eIF3i may be involved in 40S scanning (Cuchalova et al. 2010). We re-created these eIF3g and eIF3i mutations in the BY4741 genetic background (Supplemental Fig. S13). When compared to BY4741, in the temperature-sensitive *tif35-KLF* strain, GFP expression decreased two- to 2.5-fold in all ^50-500^NUS1 RNA-ID constructs irrespective of 5′ UTR length (Fig. 6A), showing no evidence of eIF3g involvement in 40S scanning. Translation on the ^122^Struct mRNA was reduced 1.5-fold, similar to translation of mRNAs with unstructured 5′ UTRs (Fig. 6A).

Amino acid substitution Q258R in eIF3i affected translation of GFP mRNAs with shorter unstructured 5′ UTRs somewhat more significantly than translation of mRNA with longer 5′ UTRs: GFP expression on the ^50^NUS1 and ^500^NUS1 mRNAs decreased four and two times, respectively (Fig. 6B). In contrast to the moderate reduction of GFP synthesis on mRNAs with unstructured 5′ UTRs, Q258R substitution in eIF3i diminished translation of the ^122^Struct mRNA 15-fold (Fig. 6B). Hence, eIF3i may be involved in the unwinding of secondary structure in 5′ UTRs rather than 40S movement along mRNA itself.

### Translational helicases Ded1 and Slh1 are not involved in 40S movement along the 5′ UTR

**FIGURE 7.**
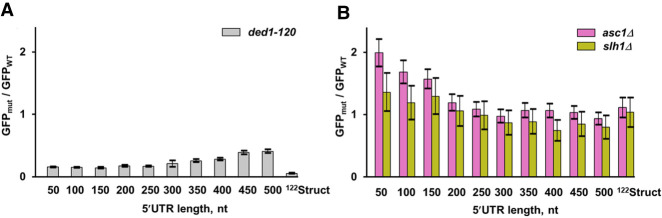
Translational helicases Ded1 and Slh1 are not essential for 40S scanning. GFP fluorescence measured in mutant strains normalized to respective GFP fluorescence in the wild-type (BY4741) strain. Error bars indicate standard deviations determined from three biological replicates. (*A*) NUS1 RNA-ID constructs were transformed into a *ded1-120* strain containing cold-sensitive allele *ded1-120*, which encodes the mutant form of Ded1 helicase bearing the amino acid substitutions G108D and G494D. (*B*) NUS1 RNA-ID constructs were transformed into the *slh1*Δ or *asc1*Δ strains, helicase Slh1 and ribosomal protein Asc1, respectively. In *A*, GFP synthesis was induced concurrently with the temperature shift from 30°C to 18°C.

We next examined involvement of two translational helicases Ded1 and Slh1 in 40S movement along the 5′ UTR. Ded1 (yeast ortholog of mammalian DDX3X) is a DEAD box helicase that is implicated in unwinding stable stem–loops in 5′ UTRs during translation initiation (Sharma and Jankowsky 2014; Sen et al. 2015). We transformed RNA-ID constructs into a yeast strain containing the cold-sensitive allele *ded1-120* (Supplemental Fig. S13), which encodes Ded1 bearing amino acid substitutions G108D and G494D (Sen et al. 2015). These mutations are thought to impair ATP binding or hydrolysis (Linder and Jankowsky 2011). GFP expression in the cold-sensitive *ded1-120* strain (Supplemental Fig. S13) was induced by the addition of galactose simultaneously with the temperature shift from 30°C to 18°C. When compared to GFP expression in the BY4741 strain at 18°C, translation of all reporter mRNAs with unstructured 5′ UTRs in *ded1-120* strain was reduced (Fig. 7A). Contrary to the idea of Ded1 involvement in 40S translocation during scanning (Berthelot et al. 2004), mRNAs with shorter unstructured 5′ UTRs were more significantly affected as GFP expression on ^50^NUS1 and ^500^NUS1 mRNAs decreased seven- and threefold, respectively (Fig. 7A). Similar results were obtained with ^50-500^NUS′ reporters (Supplemental Fig. S11E).

Consistent with the importance of Ded1-mediated unwinding of mRNA secondary structure (Sharma and Jankowsky 2014; Sen et al. 2015; Gupta et al. 2018), translation of the ^122^Struct mRNA containing a structured 5′ UTR diminished nearly 20-fold in *ded1-120* cells (Fig. 7A). Taken together, our data support the model suggesting that Ded1 is critical for melting secondary structures but not the actual movement of the 40S along the 5′ UTR.

Recently, another translational helicase, ski2-like ASCC3, was linked to 40S scanning, as ASCC3 knockdown lowered translational efficiency and 40S occupancy along the 5′ UTR in mammalian cells (Kito et al. 2023). ASCC3 and its yeast ortholog Slh1 are known to disassemble stalled 80S ribosomes in the ribosome-associated quality control (RQC) pathway (Filbeck et al. 2022). Noteworthy, ASCC3 was shown to be a 3′-to-5′ translocase, that is, it moves in the direction opposite to scanning 40S subunit (Dango et al. 2011; Best et al. 2023). Nevertheless, we tested whether yeast Slh1 may be responsible for the 40S movement along the 5′ UTR by deleting the single nonessential gene encoding this helicase. Because Slh1 interacts with nonessential protein Asc1 on the small ribosomal subunit (Best et al. 2023), we also expressed GFP reporters in *asc1*Δ strain. Relative to BY4741 wild-type cells, GFP expression of the ^50^NUS1 construct increased 1.4- and twofold in *slh1*Δ and *asc1*Δ strains, respectively (Fig. 7B). Neither genetic perturbation substantially affected GFP expression with long 5′ UTRs. Deletions of *slh1* and *asc1* similarly affected the ^50-500^NUS′ reporters (Supplemental Fig. 11F). Hence, our data provide no supporting evidence for Slh1 involvement in 40S scanning.

## DISCUSSION

Our experiments indicate that extending the unstructured 5′ UTR 10-fold over the median length in yeast transcripts reduces GFP synthesis by only approximately twofold (Fig. 3). Hence, 40S scanning is not rate limiting in yeast cells under normal growth conditions. In contrast to unstructured sequences, cap-distal elements of secondary structure in the 5′ UTR were shown to inhibit translation in our (Fig. 3) and other studies (Pelletier and Sonenberg 1985; Kozak 1986, 1989a; Vega Laso et al. 1993; Babendure et al. 2006; Sen et al. 2015). Hence, 5′ UTRs are relatively short in yeast and other taxonomic classes of eukaryotes because lengthening of 5′ UTR sequences increases the number of base pairs and thus hinders 40S scanning, as was previously suggested (Akirtava et al. 2025). The extent of secondary structure likely causes negative correlation between 5′ UTR length and gene expression levels (Vassilenko et al. 2011; Lin and Li 2012).

Undoubtedly, in addition to RNA secondary structures, other features of 5′ UTRs affect initiation efficiency. For example, upstream ORFs (uORFs), which were not present in RNA-ID 5′ UTRs, were estimated to explain ∼50% of variance in expression between different yeast 5′ UTRs (Akirtava et al. 2025). Furthermore, a number of translation enhancing and repressive sequence motifs were identified in yeast mRNAs (Niederer et al. 2022; Akirtava et al. 2025). Indeed, the NUS′ set of sequences, which lack secondary structure and uORFs, do contain two previously identified repressive motifs UCC and ACCAC (Niederer et al. 2022) and do exhibit length-dependent reduction in GFP. We infer that these effects are due to accumulation of inhibitory UCC and ACCAC motifs as the NUS1 and NUS2 set of sequences that are devoid of these repressive motifs (Niederer et al. 2022) do not exhibit length-dependent reduction in expression. Nevertheless, A-rich sequences and the AUA motif, which were shown to mildly enhance translation (Niederer et al. 2022; Akirtava et al. 2025), are present in the NUS1 and NUS2 set of sequences. Longer NUS1 and NUS′ sequences are enriched with these putative translational enhancers. This could explain why reporters with 450–500 nt-long 5′ UTRs are translated more effectively than reporters with 300–400 nt-long 5′ UTRs (Figs. 3, 5B, 7A; Supplemental Fig. 11).

Using RNA-ID reporters and mutagenesis, we attempted to identify translation factors essential for scanning. Loss-of-function mutations or deletion of eIF4B, eIF4G, eIF3g, eIF3i, Asc1 as well as translational helicases eIF4A, Ded1, and Slh1, similarly affected translation of GFP reporters with short and long unstructured 5′ UTRs, indicating that these factors are not involved in 40S scanning (Figs. 5–7). Consistent with previous observations (Sen et al. 2015; Yourik et al. 2017), the loss-of-function mutation of eIF4A equally reduced translation reporters with structured and unstructured 5′ UTRs (Fig. 5A,B). These results support the idea that eIF4A mediates recruitment of the eIF4F complex and 40S subunit to the 5′ cap rather than unwinding of mRNA secondary structure (Feoktistova et al. 2013; Sokabe and Fraser 2017; Yourik et al. 2017; Gentry et al. 2025). Interestingly, Ded1 mutation also decreased translation of reporters with unstructured 5′ UTRs (Fig. 7A; Supplemental Fig. S11E), which agrees with data linking Ded1 to the 40S recruitment step of translation initiation (Sen et al. 2015; Gao et al. 2016; Gupta et al. 2018).

In line with previous reports (Sharma and Jankowsky 2014; Sen et al. 2015, 2016), Ded1 and eIF4A cofactor eIF4B more strongly reduced translation of the reporter with a structured 5′ UTR (Figs. 5A, 7A; Supplemental Fig. S11A,E). Hence, our data provide new evidence for Ded1 and eIF4B roles in translation on structured 5′ UTRs in addition to their involvement in promoting eIF4F-dependent 40S binding to the 5′ cap (Sen et al. 2015, 2016). Unexpectedly, mutation in eIF3i, which binds near the entry of the mRNA channel (Brito Querido et al. 2024), also diminished translation of the reporter with a structured 5′ UTR (Fig. 6B), indicating that this factor may be involved in unwinding of secondary structures.

The rationale for introducing mutations into eIF4A and other factors was based on several studies suggesting that 40S movement along the 5′ UTR occurs by either processive unidirectional translocation or by eIF4A-mediated biasing of bidirectional 40S diffusion toward the start codon in the Brownian ratchet mechanism of scanning (Berthelot et al. 2004; Vassilenko et al. 2011; Garcia-Garcia et al. 2015; Wang et al. 2022). All 40S translocation models, including the Brownian ratchet mechanism, entail existence of a translocase (e.g., eIF4A), which converts the energy of a chemical reaction (e.g., ATP hydrolysis) into unidirectional movement of the 40S. Scanning time, which was either measured in single-molecule experiments (Wang et al. 2022) or inferred from reporter translation in cell extracts (Berthelot et al. 2004; Vassilenko et al. 2011), was observed to scale linearly with 5′ UTR length, which is consistent with helicase-driven translocation of the 40S subunit during scanning. It is noteworthy that assessments of the 40S scanning rate drastically differed between these reports from 6 to 10 nt/sec determined from reporter translation in cell extracts (Berthelot et al. 2004; Vassilenko et al. 2011) to 100 nt/sec measured in single-molecule experiments (Wang et al. 2022). In addition, all three studies used natural 5′ UTR sequences that have significant propensities to form extensive secondary structures (Supplemental Fig. S15). Hence, in these experiments, 43S movement along 5′ UTRs was likely hindered by and conflated with unwinding of 5′ UTR secondary structures. Furthermore, in single-molecule measurements, scanning rate estimates were based on just four 5′ UTR sequences, which were between 60 and 241 nt in length (Wang et al. 2022). The relatively small number of points and fairly narrow range of lengths might complicate distinguishing between linear versus parabolic dependencies of scanning time on 5′ UTR length indicative of translocation-driven versus diffusion-based mechanisms of 40S scanning. Therefore, it remains an open question whether 40S scanning is driven by helicase/translocase or by translocase-independent diffusion.

Our data do not rule out involvement of eIF4A and Ded1 in 40S scanning. For example, it is possible that mutations in eIF4A and Ded1 affected the ability of these factors to facilitate 40S recruitment without perturbing scanning-related functions. Alternatively, these genetic perturbations did not reduce the scanning rate enough to make it limiting for translation. Nevertheless, our data are consistent with several lines of evidence showing that helicases eIF4A and Ded1 do not drive 40S movement along the 5′ UTR. Most biochemical experiments indicate that DEAD box helicases eIF4A and Ded1 do not translocate along RNA and unwind RNA secondary structure by local strand separation with limited processivity (Yang et al. 2007; Liu et al. 2008). Previous studies showed that eIF4A affects mRNA translation irrespective of 5′ UTR length and secondary structure (Berthelot et al. 2004; Sen et al. 2015; Yourik et al. 2017; Gupta et al. 2018). A Ded1 mutation was also shown to have a similar effect on reporters with shorter and longer unstructured 5′ UTRs (Sen et al. 2015). Single-molecule measurements in a yeast system reconstituted from purified components showed that the 40S scanning rate does not depend on concentrations of eIF4A and ATP (Wang et al. 2022). Experiments performed in a mammalian system reconstituted from purified components demonstrated that the 43S initiation complex can bind to the 5′ end of an mRNA and migrate along an unstructured 5′ UTR toward the start codon in the absence of eIF4A and other RNA helicases, indicating that scanning does not require a translocase (Pestova and Kolupaeva 2002; Shirokikh and Spirin 2008). Furthermore, the strong inhibition of translation initiation by 5′ UTR secondary structure observed in our experiments and many published reports (Pelletier and Sonenberg 1985; Kozak 1986, 1989a; Vega Laso et al. 1993; Babendure et al. 2006) also provides indirect evidence that 43S initiation complex does not contain a highly processive, translocating helicase.

Although our data do not rule out helicase-driven scanning, recapitulation of cap-dependent translation initiation and mRNA scanning in translation systems, which were reconstituted in vitro from purified components (Pestova and Kolupaeva 2002; Shirokikh and Spirin 2008; Wang et al. 2022), make involvement of an unidentified helicase/translocase in 40S movement along 5′ UTR very unlikely. Hence, mRNA scanning is probably a helicase-independent, diffusion-based process as previously hypothesized for the 40S movement along nonnatural unstructured 5′ UTRs (Pestova and Kolupaeva 2002).

Many experimental findings demonstrate that numerous DNA and RNA binding proteins and protein complexes search for their binding sites via one-dimensional (1D) diffusion as they slide or hop along DNA/RNA (Gorman and Greene 2008; Cui and Joo 2019; Gentry et al. 2025). When compared to 3D diffusion, 1D diffusion greatly accelerates the rate of locating the correct binding site (Riggs et al. 1970; Berg et al. 1981; Winter et al. 1981; Berg and Ehrenberg 1982). Several lines of evidence support the idea that the 40S can move along the 5′ UTR by 1D diffusion. 3′-to-5′ backtracking of the 43S initiation complex during scanning was directly observed in in vitro single-molecule microscopy experiments (Wang et al. 2022). AUG triplets placed downstream from the start codon were shown to inhibit translation initiation indicating bidirectional movement of the 43S initiation complex (Matsuda and Dreher 2006; Li et al. 2022). Transcriptome-wide ribosome profiling experiments provide additional evidence for bidirectional scanning and backsliding of the 40S subunit during translation initiation on mRNA with 5′ UTRs, which are shorter than the 45 nt-long footprint of the 43S initiation complex (Gu et al. 2021).

The lack of a putative translocase, which would drive 40S movement, implies that 1D diffusion of the 40S is not limited to nonnatural unstructured 5′ UTRs, but also occurs on all natural mRNAs. Although 1D diffusion is bidirectional by nature, 40S diffusion along the 5′ UTR may be biased toward the start codon simply by virtue of the 40S being recruited to the cap at the 5′ end of mRNA. In that, the 5′ end of mRNA provides a boundary for 1D diffusion at the beginning of scanning. Spontaneous sampling of alternative secondary structures accompanied by unfolding of short stem–loops within the 5′ UTR (Lai et al. 2018; Spasic et al. 2018) may be sufficient to enable helicase-independent 40S diffusion.

In addition to initial 40S recruitment to the 5′ cap, other factors might facilitate diffusion of the small ribosomal subunit toward the start codon. Binding of RNA helicases near the entry to the mRNA channel of the small subunit (Brito Querido et al. 2024) might favor secondary structure unwinding downstream from the scanning 40S. At the same time, spontaneous refolding of intramolecular base pairs upstream of the initiation complex might further bias 40S diffusion toward the start codon. In addition, loading of multiple 40S subunits onto the 5′ UTR might prevent backtracking of the leading 40S subunit. This mechanism resembles an earlier hypothesis, which was proposed by Marilyn Kozak to explain the observed increase in reporter translation resulting from lengthening of the 5′ UTR (Kozak 1991).

Based on our data and a massive body of published observations, we thus conclude that 1D diffusion is a parsimonious explanation of 40S movement along the 5′ UTR during mRNA scanning.

## MATERIALS AND METHODS

### Reagents

Oligonucleotides were ordered from Integrated DNA Technologies (IDT). Enzymes were from NEB. Other chemicals and standard reagents were ordered from Fisher Scientific.

### Biological resources

Yeast *S. cerevisiae* strains used in this work are listed in Table 1.

**TABLE 1.** Yeast strains used in this work

Strain	Genotype	Reference
BY4741	*S288C MATa his3- leu2- met15- ura3-*	
BY4742	*S288C MATα his3- leu2- lys2- ura3-*	
*tif1*Δ	*BY4741 tif1::hphMX*	This study
*tif2*Δ	*BY4741 tif2::hphMX*	This study
*tif1*Δ *tif2*Δ *tif1-A79V* (H5121-NSY21)	*MATa his3- leu2- ura3- met15- tif1::hphMX tif2::KanMX pSSC120 [tif1-A79V Leu2 CEN4]*	Sen et al. 2015
*tif1*Δ *tif2*Δ *TIF1* (H5120-NSY20)	*MATa his3- leu2- ura3- met15- tif1::hphMX tif2::KanMX pFJZ600 [TIF1 Leu2 CEN4]*	Sen et al. 2015
*tif3*Δ	*BY4741 tif3::kanMX*	This study
*tif4631*Δ	*BY4741 tif4631::kanMX*	This study
*tif4632*Δ	*BY4741 tif4632::kanMX*	This study
*tif4632*Δ *tif4631-L614F*	*BY4741 tif4632::kanMX tif4631-L614F::hphMX*	Re-created from Park et al. 2013
*tif4631*Δ *tif4632-M570K*	*BY4741 tif4632::kanMX tif4631-M570K::hphMX*	Re-created from Park et al. 2013
*tif35 K194A-L235A-F237A*	*BY4741 tif35-K194A-L235A-F237A::KanMX*	Re-created from Cuchalova et al. 2010
*tif34 Q258R*	*BY4741 tif34-Q258R::KanMX*	Re-created from Cuchalova et al. 2010
*ded1-120 (G108D/G494D)*	*BY4741 ded1-120::KanMX*	Sen et al. 2015
*asc1*Δ	*BY4741 asc1::HIS5*	Wolf and Grayhack 2015
*slh1*Δ	*BY4741 slh1::kanMX*	This study

### RNA-ID constructs design and preparation

A GFP reporter containing UTRs from yeast RPL41B gene was synthesized by GenScript. The GFP ORF contains an N-terminal 3C site-HA epitope-His6-encoding sequence upstream of superfolder GFP sequence. Nonrepetitive unstructured sequences (NUS) were designed using the orega tool (optimizing RNA ends with a genetic algorithm) of the RNAstructure software package (https://rna.urmc.rochester.edu/RNAstructure.html), as previously described (Lai et al. 2018). Orega evolves sequences using a scoring function that penalizes base-pair formation and encourages a diversity of sequence motifs as quantified by linguistic complexity (Trifonov 1990; Gabrielian and Bolshoy 1999). Default parameters were used for orega. By default, 1000 iterations of evolution occur, but calculations were restarted for additional sequence evolution when sequences were still predicted to have base pairs. Base-pair probabilities were estimated using the partition function in RNAstructure (Reuter and Mathews 2010).

We used the Gibson assembly method combined with conventional restriction digestion and ligation to construct RNA-ID DNAs with various sequences (^50^NUS∼^500^NUS, ^300^NUS2, ^300^NUS + SL, ^50^NUS3 + ^122^Struct, ^50^NUS4 + ^122^Struct) at the 5′ end of the sfGFP-ORF sequence. Primers and ultramer oligonucleotides, whose sequences can be found in Supplemental Tables S2–S14, were synthesized at IDT. Detailed procedures including the production of ^122^Struct are described in Supplemental Methods.

### Yeast transformation with RNA-ID reporters

BY4741, BY4742, or mutant yeast strains were transformed with RNA-IDs constructs as previously described (Brule et al. 2016). To obtain a linear DNA fragment containing the RNA-ID reporter flanked on each end by sequences homologous to *ADE2* locus, the RNA-ID plasmid was digested with StuI restrictase (NEB) and then subjected to agarose-gel purification.

To prepare competent yeast cells for transformation, yeast cells were grown in YPD liquid media to OD_600_ between 1.3 and 2.5 (log phase). For each transformation, 10 mL of cell culture was pelleted by centrifugation at 3000 rpm for 5 min. Pellets were washed twice with 1 mL of 100 mM LiOAc. Next, cells were resuspended in 110 μL of 100 mM LiOAc containing salmon sperm DNA at a concentration of 1 mg/mL, and then incubated with 300 ng of linearized DNA at 30°C for 15 min. Next, 600 μL of a LiOAc-PEG solution (8 volumes of 60% PEG 3350 to 1 volume of 1 m LiOAc to 1 volume of ddH_2_O) was added. After incubation at 30°C for 30 min, cells were mixed with 68 μL of DMSO and incubated at 42°C for 15 min. After transformation, cells were pelleted at 1500 rpm for 7 min, resuspended in 1 mL of YPD media, and incubated at 30°C for 1 h while shaking. After this recovery period, 250 μL of cells were grown on a SC-met^−^ or SC-ura^−^ plate at 30°C for 2 days. Individual colonies were streaked onto another SC-met^−^ or SC-ura^−^ plate and grown at 30°C for 2 days.

### Flow cytometry

Cells from a single colony were inoculated into 5 mL of YP media supplemented with 2% raffinose, 2% galactose, and 80 mg/L adenine and grown overnight at 30°C. An aliquot of overnight culture was diluted to a final volume of 5 mL with the same media to obtain an OD_600_ of 0.2 and then grown for 4–6 h at 30°C to OD_600_ of ∼0.8 (Dean and Grayhack 2012). Temperature-sensitive mutants were grown in YP media containing 2% raffinose and 80 mg/L adenine (i.e., without galactose) overnight at 30°C. An aliquot of overnight culture was then diluted to final volume of 5 mL, with the same media supplemented with 2% galactose to obtain an OD_600_ of 0.2, and then grown for 4–18 h at the nonpermissive temperature (either 18°C or 37°C) to an OD_600_ of ∼0.8. Five hundred microliters of the resulting cell culture was transferred to a 5 mL polystyrene round-bottom tube (Falcon) and kept on ice for an hour. Flow cytometry was performed for three independent isolates of each strain using an LSRFortessa Flow Cytometer (BD Biosciences) (Dean and Grayhack 2012). A reference strain carrying a modified RNA-ID reporter with only a 6 nt-long 5′ UTR (GO control) is used as the primary control in all flow cytometry experiments. For RNA-ID GO control, both GFP and RFP channels were adjusted to 26,000 AU. Flow cytometry data were analyzed using the Cytobank flow cytometry data analysis platform (Chen and Kotecha 2014).

### Yeast gene deletion clone generation

We created deletion strains in a BY4741 background. To create *tif1*Δ and *tif2*Δ, yeast cells were transformed with a DNA amplicon containing KanMX DNA, which encodes bacterial aminoglycoside phosphotransferase for kanamycin resistance, flanked by 500 nt-long sequences homologous to the sequences flanking either *tif1* or *tif2* ORF in yeast genome. To obtain *tif3*Δ, *tif4631*Δ, *tif4632*Δ, and *slh1*Δ, yeast cells were transformed with a KanMX-containing cassette amplified from the respective deletion strain from the single-gene deletion collection (Giaever et al. 2002) provided by Eric Phizicky (University of Rochester). Primer sets used for each amplicon are listed in Supplemental Table S3. Amplicons were agarose-gel-purified and used for transformation to BY4741 cells by the method described above. To obtain the *asc1*Δ strain, we used the YJYW17 strain (BY4741, asc1::HIS5 + pASC1-*URA3*), in which the *asc1* ORF is replaced with the HIS5 cassette and complemented with the Asc1-expressing *URA3* plasmid (Wolf and Grayhack 2015). This strain was cultured on a 5-FOA-URA-SD plate at 30°C for 2 days to eliminate the Asc1-expressing *URA3* plasmid.

### Yeast gene mutation clone generation

We produced yeast mutant clones, which contain mutations *tif4631-L614F*, *tif4632-M570K*, *ded1-120*, *tif34-Q258R*, or *tif35-K194A-L235A-F237A*, according to the method described elsewhere (Li et al. 2011). BY4741 cells were transformed with two DNA cassettes. One cassette contained the ORF of interest with respective amino acid substitutions flanked by ∼500 and ∼300 nt-long sequences homologous to the sequences flanking the 5′ and 3′ ends of the ORF in the yeast genome. The 3′ flanking sequence was followed by ∼500 nt of KanMX or HgrB DNA. The second cassette contained full-length KanMX or HgrB DNA followed by a 500 nt-long sequence homologous to the respective sequence of yeast genome positioned 300 nt downstream from the ORF of interest. These cassettes were excised by restriction enzymes from the plasmids listed in Supplemental Methods. Mutations were verified by sequencing of the respective ORFs in genomic DNA.

### Other procedures

Yeast genomic DNA (gDNA) purification, RT-qPCR for GFP and RFP mRNAs, SDS-PAGE analysis with fluorescence detection, 5′RACE analysis, and examining yeast growth via spot assay were done using standard procedures described in the Supplemental Material.

## SUPPLEMENTAL MATERIAL

Supplemental material is available for this article.
